# Methylene Blue to Neonatal Septic Shock treatment in neonate pigs^,^

**DOI:** 10.1016/j.clinsp.2022.100139

**Published:** 2022-11-24

**Authors:** Walusa Assad Gonçalves-Ferri, Agnes Afrodite Sumarelli Albuquerque, Renata Sayuri Ansai Pereira de Castro, Cristina Helena Faleiros Ferreira, Luis K. Oharomari, Diego Fernando Silva Lessa, Paulo Roberto Barbosa Evora

**Affiliations:** Faculdade de Medicina de Ribeirão Preto (FMRP), Universidade de São Paulo, Ribeirão Preto, SP, Brazil

**Keywords:** Methylene blue, Septic shock, Vasoplegic syndrome, Nitric oxide, Infants, Newborn, MB, Methylene Blue, BP, Blood Pressure, NO, Nitric Oxide, Cgmp, Cyclic Guanosine Monophosphate, ARDS, Acute Respiratory Distress Syndrome, CVP, Central Venous Pressure, MAP, Mean Arterial Pressure, MDA Malondialdehyde Acid, Spap, Systolic pulmonary artery pressure

## Abstract

**Objective:**

The authors design an animal model of neonatal sepsis to analyze the treatment of neonatal septic shock with Methylene Blue (MB) in a swine model.

**Methods:**

The study design included twenty male newborn pigs divided into four groups: 1) The control group; 2) The sepsis group (induced with lipopolysaccharide); 3) The MB group, and 4) The MB-treated sepsis group. Septic shock was defined as Blood Pressure (BP) dropping 20% below the baseline value. Continuous Blood Pressure (BP), Nitric Oxide (NO) levels, cyclic Guanosine Monophosphate (cGMP), malondialdehyde acid, base excess, lactate, arterial blood gases, hematocrit, and echocardiography were analyzed.

**Results:**

The BP of the sepsis group treated with MB showed a slight improvement in the first hour after treatment; however, a significant difference was not observed compared to the untreated sepsis group. Besides hemodynamic stability, the current study did not show symptomatic pulmonary hypertension, suggesting that MB was safe in neonates and children. An improvement in Base Excel (BE) levels after MB administration in septic animals may indicate a possible improvement in microcirculation.

**Conclusion:**

The MB improved biomarkers related to septic shock prognosis, although an improvement in the blood levels could not be detected. MB might be a beneficial drug for hemodynamic instability in infants.

## Highlights

• MB improved septic shock biomarkers associated with microcirculation, however, did not improve blood pressure.
• The MB was not associated with pulmonary hypertension and respiratory clinical repercussions.
• The MB did not decrease the heart inotropic or chronotropic aspects.

## Introduction

Methylene Blue (MB) is an optional treatment of distributive shock resulting from systemic inflammatory reaction refractory to standard measures. Methylene blue is recommended to increase blood pressure in shock by interfering with guanylate cyclase activity. Several studies have evaluated the vasoconstrictor and positive inotropic effects of MB on septic shock, anaphylactic shock, toxin-induced shock, and patients with vasoplegic syndrome following myocardial revascularization surgery.^1^, ^2^, ^3^, ^4^, ^5^, ^6^

Although its use shows a tendency toward acceptance in adults, it does not occur for its use in children. This fact is observed from both an experimental and a clinical point of view; MB in children has two main concerns: pulmonary hypertension and controversial use in Acute Respiratory Distress Syndrome (ARDS). These aspects have not been systematically studied. Few studies have reported that MB increases blood pressure and pulmonary vascular resistance. It is noteworthy that in very few studies, these elevations in pulmonary vascular indices were observed as clinically and statistically insignificant.^5^

Publications reporting the use of MB in pediatrics and neonatology generally warn of possible deleterious effects of its use in this age group, but without the clinical or experimental basis. One gets the impression even of creating a paradigm of contraindication of medication. Thus, the present investigation was based on a rational analysis that would be worth testing the hypothesis that blockade of the NO/cGMP system may be detrimental to the pulmonary circulation of neonates. It is also noteworthy that few models use a neonatal animal model with extensive experiments lasting hours.^6^, ^7^, ^8^, ^9^, ^10^

Considering that vasodilatation can play an essential role in the pathophysiology of septic shock, MB may be a beneficial therapeutic. There are few studies on newborn infants, and to our knowledge, only case reports have been published.^9^^,^^10^ Therefore, the present investigation aimed to analyze the MB treatment of neonatal septic shock in a neonatal swine model.

## Method

### Animals and drugs

Four to eight days old Camborough male pigs were brought daily from an accredited farm for breeding experimental animals. The neonate animals remained with their mother, on free food, until 1 hour before the experiment. The animal procedures as well as the adopted protocols of this study were approved by the Committee of Ethics in Animal Experimentation (CETEA) of the Ribeirão Preto Medical School (FMRP) number 23/2015. The animals were euthanased after the study through high doses of anesthetic drugs.

The models that presented some disease or pneumothoráx were excluded (1 animal /pneumothorax) ‒ the study following the ARRIVE 2.0 guideline.^11^

### Anesthetic and operative technique

Upon arrival at the laboratory, the animal received pre-anesthetic xylazine + ketamine (1:1), 1 mL/kg at the end of the experiment. The pigs were sacrificed with one over-anesthetic dose. After anesthetic induction and anesthesia, the animal was sedated with continuous midazolam infusion (0.3 mg/kg/hr) and fentanyl (3 mcg/kg/hr). The animal's breathing movements were continuously evaluated, and if spontaneous breathing was present, the sedation was increased by 30% of the initial dose.

### Ventilatory management

The animal was intubated by laryngoscopy with a cannula 3 or 3.5, fixed at 10 cm, with the position conferred through pulmonary auscultation. Ventilatory assistance was provided by a mechanical ventilator (Model IV ‒ 100B Sechrist, Anaheim, CA, USA), IVM mode, with initial parameters pi15, peep5, FR40, tins 0.35, fiO_2_ 21%. The ventilatory parameters were changed from Ph 7.3 to 7.4, pCO_2_ 40‒60, and pO_2_ 50‒60. Target saturation was 92%‒94%. The clinical ventilatory evaluation was determined by bilateral and symmetrical thoracic expansion, 1/3 of the thoracic diameter. If the need for ventilatory change and chest with adequate expansion, the respiratory rate was increased by 10 points until the desired gasometric parameters were obtained. If the peripheral saturation was outside the selected range, the oxygen supply was altered by 10%.

### Temperature control

The temperature was controlled rectally through the mercury thermometer in a thermal blanket (brand), and the objective rectal temperature was 38° to 40°C.

### Septic shock induction

Septic shock was induced by introducing LPS (Lipopolysaccharidesfrom *Escherichia coli* O111: B4 ‒ Sigma), at a dose of 0.06 mg/kg, introduced through the arterial route.

### Methylene blue administration

Methylene blue was administered to the sepsis group as 20 perpetual points fall in basal pressure was soon detected. A bolus infusion of 2 mg/kg was initially injected in a 15-minute infusion pump after 1 hour if the shock was reversed (fall lower than 20%) of baseline pressure, methylene blue was installed at the initial dose of 0.5 mg/kg/h and, the dose offered every 30 minutes was reevaluated. If after 30 minutes the animal maintained the shock, the infusion rate was increased by 0.5 mg/kg/hr, reconsidered every 30 min with the same procedure, up to a maximum dose of 2 mg/kg/hr. If the animal had mean arterial pressure values at baseline levels, continuous infusion of methylene blue would be maintained at the dose at which the pressure was reached. In the sham group, methylene blue was administered at 100 to 120 minutes of life, with the initial bolus, followed after one hour by continuous infusion as described above.

### Study design

The animals were separated according to 4 groups, following the sequence below. When the authors completed the series, then restarted to complete five animals/groups:

Control group (C): Animals were sedated and ventilated for 6 hours.

Sepsis group (S): Sedated animals, ventilated for 6 hours with LPS infusion.

Methylene Blue Group (MB): Sedated animals, ventilated for 6 hours with the administration of methylene blue.

Methylene blue treated sepsis group (SMB): Sedated animals, ventilated for 6 hours, infused with LPS, and administration of methylene blue.

The study design did not permit blindness.

### Hemodynamic study

Venous and arterial access was obtained by desiccation and cannulation of the left femoral vein, left femoral artery, and right jugular vein. A catheter was introduced through the left femoral vein to monitor Central Venous Pressure (CVP). Another polyethylene catheter was introduced into the left femoral artery to obtain Mean Arterial Pressure (MAP). The SBP monitoring was performed using the System MP 100 A (Biopac System, Inc., Santa Barbara, CA, USA) connected to a PC Gateway (Gateway, Sioux City, SD, USA) with Windows XP operating system, which can collect, analyze, store and retrieve biophysical data. The vascular catheters were connected to pressure transducers, and these, were to the continuous registration MP System 100 A. The authors performed volume replacement (10 mL/kg of isotonic solution) if the blood samples collected were more than 20% of the animal's estimated blood volume (8% weight).

### Blood gas analysis

The biochemical measurements of pH, partial carbon dioxide (pCO_2_) arterial pressure, and plasma concentration of bicarbonate ion (HCO_3_-) were performed by a previously calibrated Hemogasometria Gem Premier 3000 (Instrumentation Laboratory Co., Bedford, Massachusetts, USA) using the QM 150 GEM Premier iQM Instrumentation Laboratory Co., Bedford, Massachusetts, USA.

### Nitrite (NO_2_-) and Nitrate (NO_3_-) analysis

Nitrite (NO_2_-) and Nitrate (NO_3_-) analysis were measured by a femoral artery blood sample and placed in a heparinized tube. Plasma was obtained by centrifugation at 2500g (10 minutes, 4°C) and immediately immersed in liquid nitrogen, and stored in a freezer (-70°C) for subsequent dosing of plasma NO_2_-/NO_3_. Aliquots of 25 μL of plasma were deproteinized by incubation with absolute ethanol (4°C), maintained for 30 minutes in a freezer (-20°C), and then centrifuged at 4000g for 10 minutes (Eppendorf centrifuge 5810R, Hamburg, Germany) for further dosing (NO_3_) was measured using the Sievers® Nitric Oxide Analyzer 280 analyzer (GE Analytical Instruments, Boulder, CO, USA).

### Determination of the malondialdehyde (MDA)

The colorimetric determination of MDA by its reaction with thiobarbituric acid was performed at 532 nm on a Versamax (Molecular Devices) microplate reader using 1,1,3,3-tetra methoxy propane (0–100 μM) as standard, and the results obtained were expressed in μM/mg of protein.

### Statistical analyses

Statistical analysis for SBP was the analysis of variance by two-way ANOVA followed by Bonferroni post-test; the indirect measurements of NO plasma analysis were one-way ANOVA. The software used was GraphPad Prism version 5.0 (GraphPad Software Corporation, La Jolla California, USA). The level of significance adopted was p < 0.05.

## Results

The sample size was 20 piglets. The average weight of the animals was 2391,2g, and the average lifetime was six days of life.

### Blood pressure levels

**Figure 1:**
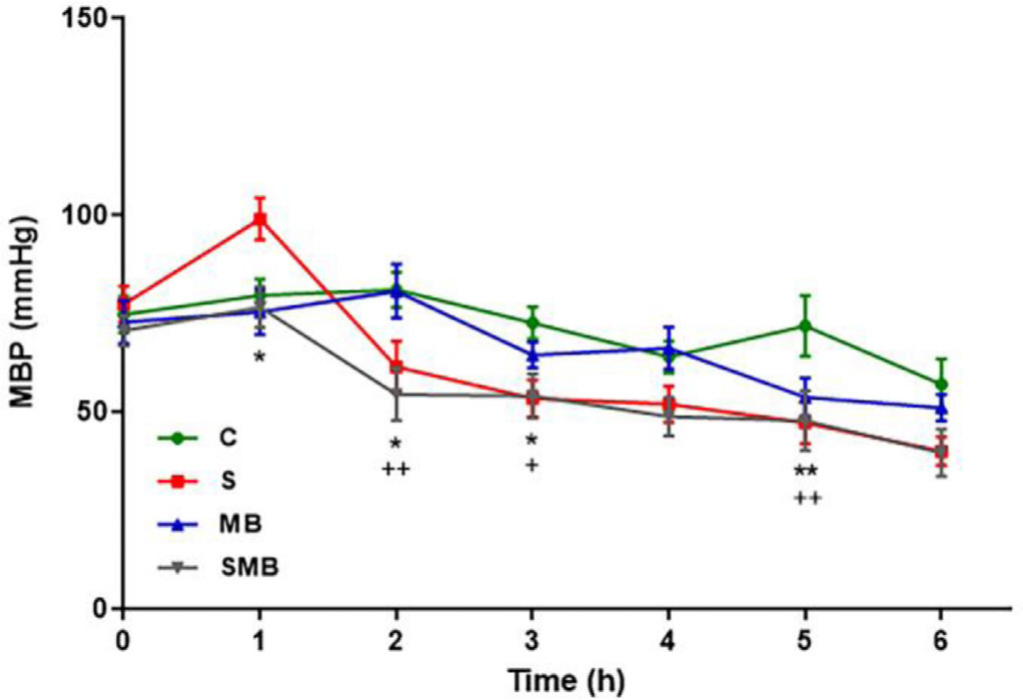
Effect of septic shock treated/ or not with Methylene Blue (MB) in neonatal pigs. Mean Blood Pressure (MBP). Data represent means ± standard error of mean and analyzed by two-way analysis of variance (ANOVA), Dunnett's post-test (n = 5). (*p < 0.05; **p < 0.01).

**Figure 2:**
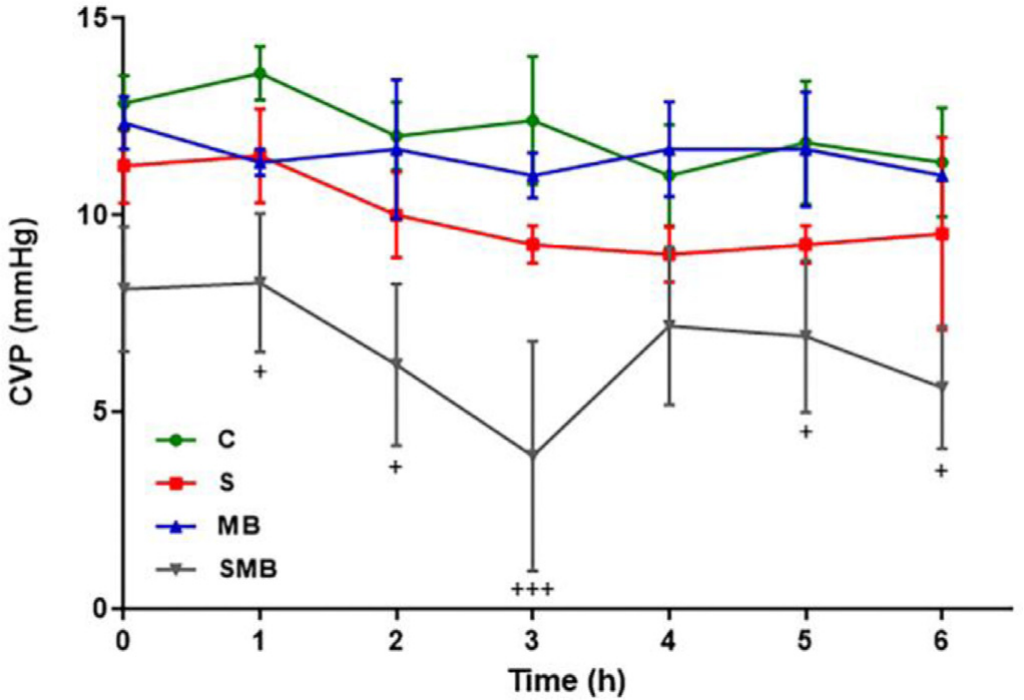
Effect of septic shock treated or not with Methylene Blue (MB) in neonatal pigs. Central Venous Pressure (CVP). Data represent means ± standard error of mean and analyzed by two-way analysis of variance (ANOVA), Dunnett's post-test (n = 5).

The C group maintained hemodynamic stability and was used as a reference. The MB group did not present a difference when compared with controls, showing the safety of the drug. The S group initially rose in the first 2 hours and then showed a significant drop. The MB was administrated at 90 minutes of the experiment, and the SMB group present a significant decrease in the blood pressure variables, during the analysis (Fig. 1). Interestingly, the SMB group was the unique group that showed a significant difference in CVP values (Fig. 2).

### Hemoglobin levels

The initial hemoglobin levels in the first hour of the experiment were, for the control group: 26 g/dL (SD = 2.9), methylene-blue 28 g/dL (SD = 1.7), sepsis: 17 g/dL (3.9), and sepsis treated with methylene blue: 28 (SD = 5.7), respectively; in the sixth hour, the values were respectively according to the group 16 g/dL (2.4), 18.7 (SD = 4.5), 17 g/dL (3.9) and 20 g/dL (SD = 5.1).

### Lactate, bicarbonate, and base excess (BE) levels

**Figure 3:**
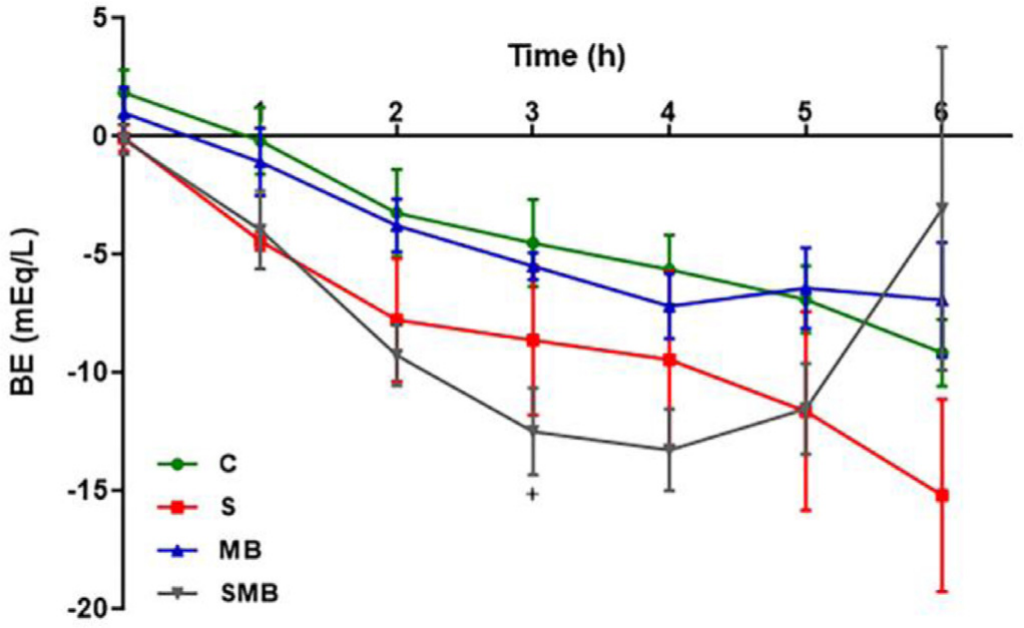
Effect of septic shock treated/ or not with Methylene Blue (MB) in mean values of Base Excess (BE) in neonatal pigs. Data represent means ± standard error of mean and analyzed by two-way analysis of variance (ANOVA), Dunnett's post-test (n = 5). (*p < 0.05; **p < 0.01; ***p < 0.001; ****p < 0.0001; C vs. S: *, C vs. SMB: +).

In the C and MB groups, the BE levels remained constant. The S and SMB groups exhibited a decrease in BE. After MB infusion, however, the SMB group showed a tendency to recover as BE values normalized within 3 hours into the experiment (Fig. 3).

**Figure 4:**
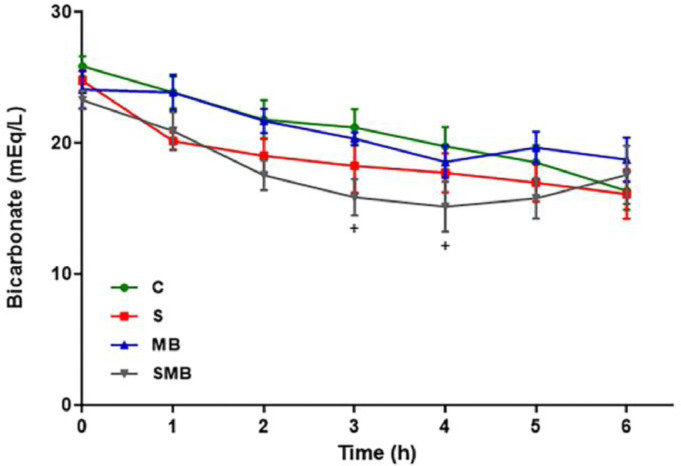
Effect of septic shock treated/ or not with Methylene Blue (MB) in mean values of blood bicarbonate (HCO_3-_) in neonatal pigs. Data represent means ± standard error of mean and analyzed by two-way analysis of variance (ANOVA), Dunnett's post-test (n = 5). (*p < 0.05; **p < 0.01; ***p < 0.001; ****p < 0.0001; C vs. S: *, C vs. SMB: +).

Lactate levels also remained constant in the C and MB groups but significantly increased in the S and SMB groups. Initially, the bicarbonate levels showed low variability between groups. Still, they were substantially more down in the SMB group between the 3 and 4 hours, increasing to normal levels at the 5 and 6 hours (Fig. 4).

### Fluid therapy

The C group did not require volume expansion to maintain intravascular volume. Only one animal expansion was needed in the MB group. Respectively, in the S and SMB groups, 2 and 3 animals required volume expansion, respectively. Fluid therapy for all the animals, as mentioned above, was required 5 hours into the experiment. There was a slight decrease in the PaO_2_/FiO_2_ ratio in the SMB group, but it remained within the normal range.

### Nitrite and nitrate

A significant difference in NO levels was observed between the C and S groups, C and MB groups, C and SMB groups, and MB and SMB groups. There was an increase in the NO_3_- levels in the S group compared to the other groups during the entire study period. With a marked increase in the last hours of the experiment (between 5 and 6 hours). In the SMB group, NO production was inhibited by MB after the first hour (between 1 and 2 hours), and partial inhibition was observed in subsequent hours (3 to 6 hours). An increase in NO_2_- levels of the S group was detected only in the last hours of the experiment (4 to 6 hours). In the SMB group, it was possible to observe complete inhibition of NO production during septic shock with MB treatment.

### Malondialdehyde acid (MDA)

**Figure 5:**
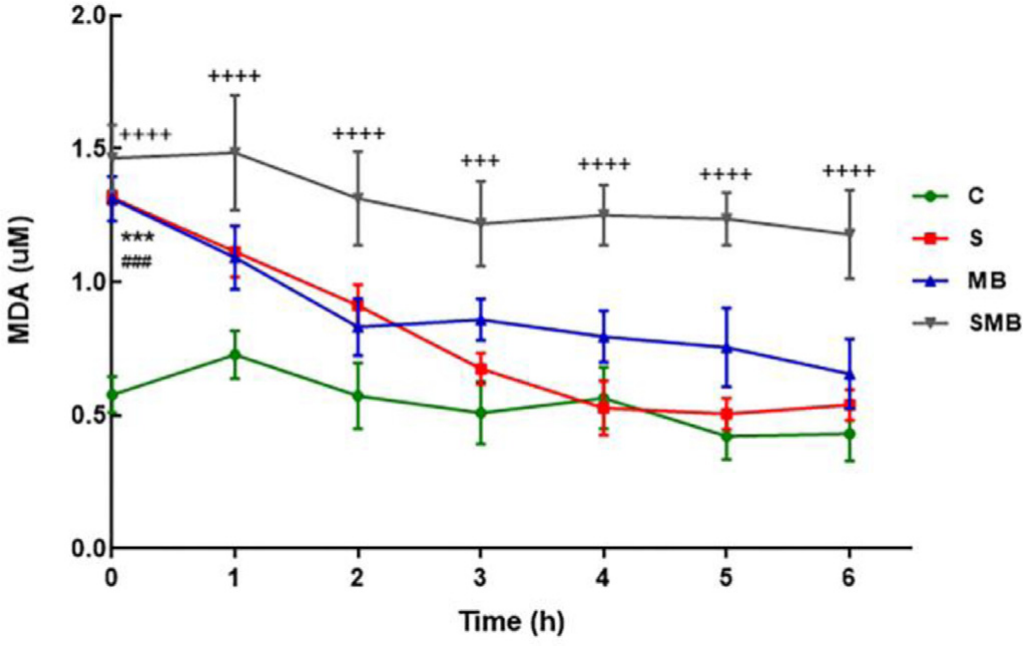
Effect of septic shock treated or not with Methylene Blue (MB) in Malondialdehyde Acid (MDA) levels. in neonatal pigs. Data represent means ± standard error of mean and analyzed by two-way analysis of variance (ANOVA), Dunnett's post-test (n = 5). (***p < 0.001; ****p < 0.0001; C vs. S: *, C vs. MB: #; C vs. SMB: +).

Significant initial differences in MDA levels were observed between the C and S groups and the C and MB groups. The C and SMB groups showed statistical differences at 0, 3, 4, 5, and 6 hours of the experiment (Fig. 5).

### Echocardiography

There were no marked differences in the animal groups' systolic and diastolic ventricular function and myocardial contractility. The following parameters did not show significant variation between the groups: End-systolic and end-diastolic diameters of the left ventricle; Ejection and shortening fraction; MAPSE; E/A wave velocity ratio; E/E' wave velocity ratio.

However, increased systolic Pulmonary Artery Pressure (sPAP) was observed in the SMB group, the average sPAP was 33.35 mm.Hg at baseline, 34.6 mm.Hg after septic shock, and 50.46 mm.Hg after MB treatment. Unfortunately, when there was no tricuspid insufficiency, it was impossible to measure sPAP and compare the sPAP levels of the different groups (Table 1).

**Table 1:** Echocardiographic evaluation of the sepsis (PS), methylene blue (PA) and methylene blue + sepsis/ treatment group (PSA).

ECHO	PA		PS		PSA		
	Base line	After MB	Base line	Shock	Base line	Shock	After MB
LVED	1.56	1.4	1.66	1.1	1.7	1.41	1.22
LVES	0.96	0.51	1.18	0.75	1.13	0.9	0.89
LVEF	72	76.5	59	65	65.3	69	71.6
LVSF	38.1	41.9	28.9	31.8	33.2	35.7	34
E wave	41.2	39.5	44.1	38.2	53.4	137.4	47.5
A wave	48.5	56.8	55.4	45.5	63.9	48.3	57.7
E/A	0.87	0.7	0.8	0.84	1.85	0.94	0.81
MAPSE	0.45	0.44	0.49	0.26	0.47	0.37	0.27
e'	3.25	3.05	2.07	X	3.07	2.6	8.71
E/e'	12.7	12.9	21.3	X	17.2	17.3	15.5
IVCd	0.36	0.33	0.38	0.16	0.46	0.36	0.28
CVCC	16	19.5	23	100	25.6	32.6	27.6
PASP	X	37.3	X	X	33.5	34.6	50.46

Echocardiographic evaluation of the Sepsis group (S), Methylene Blue group (MB), and Methylene Blue + Sepsis treatment group (SMB). LVED, Left Ventricular End-Diastole Diameter; LVES, Left Ventricular End-Systole Diameter; LVEF, Left Ventricular Ejection Fraction; LVSF, Left Ventricular Shortening Fraction; E and A wave, Peak velocity blood flow from left ventricular relaxation in early diastole and peak velocity flow in late diastole; MAPSE, Mitral Annular Plane Systolic Excursion; E/E', An estimate of left atrial filling pressure; PASP, Pulmonary Artery Systolic Pressure; X, The parameter was not possible to measure.

## Discussion

MB treatment in pediatric and neonatal patients has not been well established. Only 10 case reports and one non-randomized clinical trial were found.^6^^,^^9^^,^^10^^,^^12^, ^13^, ^14^, ^15^, ^16^, ^17^, ^18^, ^19^, ^20^ Of these, only two reported cases of newborn infants.^9^^,^^10^

The neonatal model of septic shock and methylene blue proposed is feasible. Thus, studies with this medication in the neonatal period may be promising. To the best of our knowledge, there is no newborn animal model that describes the effects of MB on septic shock.

The effects of MB treatment on blood pressure remain controversial; a rat model that used MB to treat sepsis observed higher MAP levels in the MB-treated group, in addition to better respiratory function and delayed mortality.^21^ Another animal study demonstrated that only a combined treatment of MB and norepinephrine could restore MAP levels.^22^ The blood pressure did not differ significantly between the groups in this study.

The diagnosis of shock goes beyond BP levels. Therefore, evaluating other biomarkers is mandatory. The present study showed that BE levels improved after initial worsening due to LPS infusion during MB infusion. The authors observed a similar pattern in the lactate levels. Both BE and lactate levels are associated with adverse outcomes.^23^, ^24^, ^25^ Lactate levels increased after MB administration in septic animals was registered. It is important to note that MB can react directly with lactate. Therefore, its decrease does not necessarily mean improvement in tissue perfusion.

MB acts by inhibiting endothelial Nitric Oxide (NO) (265), and its action on microcirculation may explain the improvement in BE levels. Nantais et al.^22^ used intravital microscopy to demonstrate the effect of MB on the microcirculation during septic shock. A significant decrease in leukocyte adhesion and improved functional capillary density was observed, indicating that microvasculature function had improved. Ratiani et al. showed an association between altered microcirculation in sepsis and poor prognosis.^26^,^27^ This review related microvascular dysfunction to, among others, dysfunction of vascular autoregulatory mechanisms by NO. Therefore, NO inhibition may be a desirable effect of MB treatment in patients with sepsis.

Regarding the nitrate dosage, there was a tendency to increase the NO_3_ values in the sepsis group compared to the other groups at all studied times (0 to 6 hours), and significant differences were observed between the S and MB groups and between the S and SMB groups. Therefore, the MB favorable effect was better demonstrated by nitrate dosage.

Malondialdehyde (MDA) is an important marker of oxidative stress, where increases in plasma MDA may result from sepsis-induced oxidative stress. The MB and SMB groups showed increased values compared to the control group. This result was unexpected as several studies on ischemia-reperfusion showed a protective effect of MB, where a reduction in reactive oxygen species produced in liver tissue was observed. This effect has been observed in studies that administered MB before and after ischemia and before reperfusion.^4^^,^^13^^,^^28^ Perhaps the sequence and timing of the MB treatment used in this study could not detect the reducing effect of MB on MDA levels.

Despite an increase in the systolic pulmonary artery pressure observed in the echocardiographic measurements, any group had no increased oxygen requirement. The PaO_2_/FiO_2_ ratio remained within normal ranges.

There are few studies regarding systolic pulmonary artery pressure in neonates treated with MB. About the neonate case reports, even though they did not document the echocardiographic values clinically, the authors can note that there were no variations in oxygen requirement after MB treatment, being an indirect signal of systolic pulmonary artery pressure stability.^9^^,^^10^

In addition, Evgenov et al. reported the effects of MB treatment in an endotoxemia study using sheep. After MB treatment, the study showed endotoxin-induced pulmonary hypertension and edema attenuation.^29^ Galili et al. also showed a decrease in alveolar damage in septic rats who received MB treatment.^21^ However, the data are not enough to conclusions, so the effects of MB on systolic pulmonary artery pressure need to be further studied.

Leyh et al. reported a decrease in cardiac output after the infusion of MB in adults with post-cardiopulmonary bypass-induced norepinephrine-refractory vasoplegia.^30^ However, adult animal models did not show the same effect on cardiac output,^31^^,^^32^ consistent with the echocardiographic findings regarding systolic and diastolic ventricular function. MB did not affect the myocardial contractility in the present study.

The volumes of blood collected impact the hemoglobin levels. However, the authors consider that it added a substantial translational result since septic neonates in the NICUs frequently present low Hemoglobin (Hb) levels. Also, the decrease in Hb levels was the same in all studied groups.

One limitation of the present study was that the protocol required large volumes of blood samples. However, given the same DBP monitored between the SMB group and S, MB groups, the authors believe there was no significant hypovolemia during the experiment. Also, the authors did not evaluate renal function, and cardiac and liver enzymes, which could lead to a more precise hemodynamic instability characterization.

The roles of the l-arginine and NO pathways in pediatric and neonatal patients are well documented.^18^, ^19^, ^20^, ^21^, ^22^, ^23^, ^24^, ^25^, ^26^, ^27^, ^28^, ^29^, ^30^, ^31^, ^32^ Nevertheless, research on MB treatment in these patient age groups is scarce. There is no substantial evidence that contraindicates the use of MB. Therefore, MB is a potentially beneficial drug for neonatal hemodynamic management since it can protect microcirculation, so further investigation should be done on MB use.

## Conclusion

MB improved septic shock biomarkers, albeit no significant difference in blood pressure levels can be detected. The MB was not associated with pulmonary hypertension and respiratory clinical repercussions.

## Authors’ contributions

Walusa Assad Gonçalves Ferri: Study design, planning, execution, coordinator, writer, and investigator principal.

Agnes Afrodite Sumarelli Albuquerque: Execution, planning, collecting the data, reviewer.

Renata Sayuri Ansai Pereira de Castro: Reviewer, collected the data.

Cristina Helena Faleiros Ferreira: Reviewer, and writer, collected the data.

Luis K Oharomari Jr.: Reviewer, intellectual collaboration.

Diego Fernando Silva Lessa: Reviewer, collected the data, coordinator of Echocardiographic evaluation.

Paulo Roberto Barbosa Évora: Planning, coordinator of surgical management, writer, and reviewer.

## Declaration of Competing Interest

The authors declare no conflicts of interest.
